# Spatial and geometric learning for classification of breast tumors from multi-center ultrasound images: a hybrid learning approach

**DOI:** 10.1186/s12880-024-01307-3

**Published:** 2024-06-05

**Authors:** Jintao Ru, Zili Zhu, Jialin Shi

**Affiliations:** 1 Department of Medical Engineering, Shaoxing Hospital of Traditional Chinese Medicine, Shaoxing, Zhejiang People’s Republic of China; 2 Department of Radiology, The First Affiliated Hospital of Ningbo University, Ningbo, Zhejiang People’s Republic of China; 3 Rehabilitation Medicine Institute, Zhejiang Rehabilitation Medical Center, Hangzhou, Zhejiang People’s Republic of China

**Keywords:** Breast tumor classification, Ultrasound images, Federated learning, Convolutional neural network, Graph neural network, Artificial intelligence

## Abstract

**Background:**

Breast cancer is the most common cancer among women, and ultrasound is a usual tool for early screening. Nowadays, deep learning technique is applied as an auxiliary tool to provide the predictive results for doctors to decide whether to make further examinations or treatments. This study aimed to develop a hybrid learning approach for breast ultrasound classification by extracting more potential features from local and multi-center ultrasound data.

**Methods:**

We proposed a hybrid learning approach to classify the breast tumors into benign and malignant. Three multi-center datasets (BUSI, BUS, OASBUD) were used to pretrain a model by federated learning, then every dataset was fine-tuned at local. The proposed model consisted of a convolutional neural network (CNN) and a graph neural network (GNN), aiming to extract features from images at a spatial level and from graphs at a geometric level. The input images are small-sized and free from pixel-level labels, and the input graphs are generated automatically in an unsupervised manner, which saves the costs of labor and memory space.

**Results:**

The classification AUC_ROC_ of our proposed method is 0.911, 0.871 and 0.767 for BUSI, BUS and OASBUD. The balanced accuracy is 87.6%, 85.2% and 61.4% respectively. The results show that our method outperforms conventional methods.

**Conclusions:**

Our hybrid approach can learn the inter-feature among multi-center data and the intra-feature of local data. It shows potential in aiding doctors for breast tumor classification in ultrasound at an early stage.

## Introduction

The incidence of breast cancer ranks first among females in the cancer statistics and still keeps increasing. Although the mortality is decreasing, breast cancer is still a great threat to women’s health [1]. Early diagnosis and proper treatment can improve the quality of life for patients. The ultrasound as one of common tools for early screening breast cancer has advantages of non-invasion, non-radiation, etc [2]. The ultrasound waves transmitted by the probe can penetrate biologic tissues, images are obtained while the processor processes echoes received by the probe [3]. According to the ultrasound images, doctors will diagnose the breast lesion as benign or decide to make further examinations such as biopsy.

In recent years, artificial intelligence has developed rapidly and is expected to be an auxiliary tool for doctors in disease diagnosis [4, 5]. CNN relying on its advantages in the domain of image processing has been widely used in tumor detection, segmentation, classification, etc. The aim of detection is to find bounding boxes around tumors. In [6] researchers combined CNN and gated recurrent units to detect invasive ductal carcinoma in pathological images. In [7], a ShuffleNet-ResNet scheme was proposed to detect breast cancer in mammograms and ultrasound datasets. An annotation-efficient deep learning approach in [8] was designed for cancer detection in digital breast tomosynthesis. Unlike detection, segmentation will produce a pixel mask that yields shaped contours [9]. embedded a spatial-temporal transformer in encoder-decoder layers for breast tumor segmentation in DCE-MRI. A global guidance network [10] was proposed for breast lesion segmentation in ultrasound images, aiming to capture long-range dependencies of the inputs and improve lesion segmentation accuracy. Researchers in [11] used attention modules to guide a neural ordinary differential equation based framework to segment breast tumors in ultrasound and DCE-MRI, alleviating the problems such as large amounts of parameters, lack of interpretability, overfitting problem, etc. Since ultrasound images are vulnerable to speckle noise interference [12, 13], researchers in [14] studied the segmentation of breast tumors from ultrasound images with different kinds of despeckling algorithms. In [15], a Hybrid-UNet which created using SegNet and UNet was proposed to segment thyroid tumors from despeckling ultrasound images. For classification [16], proposed a multi-DCNN framework to classify breast cancer in mammograms. In [17], the researchers added segmentation-based attention block to the deep CNN for breast tumors classification in ultrasound which was a segmentation-classification scheme. In [18], the authors designed an automatic classification model for histopathological images based on deep feature fusion and enhanced routing. In [19], edge preserving smoothing despeckling filter and encoder- decoder-based ResNet50 segmentation model were used for ultrasound images at preprocessing stages. Then the researchers extracted information from thyroid tumor by fifteen deep learning-based pretrained models and finally trained PCA-SVM for classification.

Images are Euclidean data that of translation invariance. Unlike images, graphs are non-Euclidean data which can be visualized as aggregations of nodes and edges without having any order. The advent of GNN has provided a powerful technique to process graph data by exploiting the node relationships [20]. In [21], a fingerprints-GNN was proposed to predict molecular properties of breast cancer [22]. used graph representations of the cellular interconnection geometry in a whole slide image to predict HER2 status in breast cancer. In [23], a hierarchical Graph V-Net was designed to classify histopathological images.

The combination of these two networks has also been explored by researchers. In [24], the authors extracted features by CNN to construct graphs and then used GNN for automatic characterization of both the morphology and distribution of microcalcifications in mammograms. Researchers in [25] used a CNN to extract features from DCE-MRI scans and an autoencoder to represent genomic variant results or micro array expression features in a condensed latent space. The combination of radiographic data and genomic data improved the GNN abilities for prediction of breast cancer molecular subtype.

The combination of CNN and GNN for classification of breast cancer in ultrasound is rarely studied. Thus, in this study we designed a hybrid learning architecture that contained CNN and GNN to achieve spatial learning, geometric learning and federated learning simultaneously, aiming to make better use of multi-center ultrasound images and protect privacy at the same time.

The main contributions of our work are as follows:

1. A hybrid learning approach consisting of federated learning, spatial learning and geometric learning was firstly proposed for breast tumor classification in multi-center ultrasound data;
2. The images did not need doctors to delineate the contours of tumor in advance, and the graphs were generated from images automatically in an unsupervised manner;
3. The federated learning was used to extract inter-feature among multi-center ultrasound without data exchange and privacy leakage;
4. The CNN branch was designed to extract features in spatial domain from small sized images, while the GNN branch was designed to extract features in geometric domain from graphs. Intra-feature extracted from two branches was fused and classified using a multi-layer perceptron (MLP) finally.

The rest of this article is organized as follows: all the datasets and techniques we used are presented in Section “Materials and methods”; Evaluation metrics and experimental results are shown in Section “Results”; Section “Discussion” gives a comprehensive discussion on our method and comparative methods according to the experimental results and describes future work; Section “Conclusions” concludes the article.

## Materials and methods

### Ultrasound datasets

#### BUSI

This dataset was collected by Baheya hospital, Egypt [26]. It consists of breast ultrasound images (*n* = 780) of 437 benign cases, 210 malignant cases and 133 normal cases with confirmed pathological diagnosis. These images were scanned by LOGIQ E9 ultrasound system and LOGIQ E9 Agile ultrasound system ML6-15-D Matrix linear probe transducer (1–5 MHz).

#### BUS

It is a public breast ultrasound dataset that was collected from the UDIAT Diagnostic Centre of the Parc Tauli Corporation, Sabadell (Spain) [27]. Within the dataset (*n* = 163), there are 53 malignant cases and 110 benign cases with confirmed pathological diagnosis. These images were scanned with a Siemens ACUSON Sequoia C512 system 17L5 HD linear array transducer (8.5 MHz).

#### OASBUD

The free available ultrasonic radio-frequency (RF) echoes were recorded from breast lesions in the Department of Ultrasound, Institute of Fundamental Technological Research Polish Academy of Sciences in Warsaw [28]. These RF signals were obtained by the Ultrasonix SonixTouch Research ultrasound scanner using the L14-5/38 linear array transducer (10 MHz). The dataset (*n* = 200) contains longitudinal and transverse scans for each case, so there are 104 malignant samples and 96 benign samples with confirmed pathological diagnosis.

**Fig. 1:**
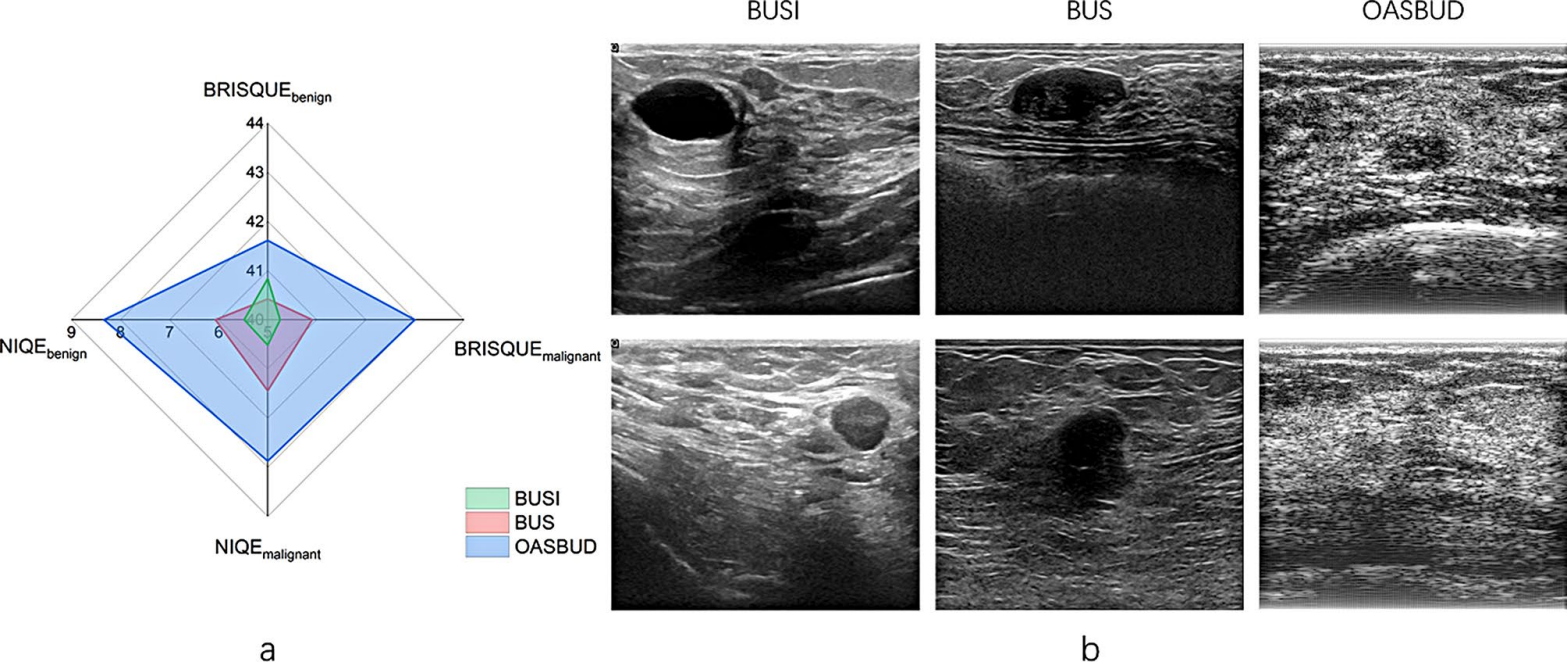
**(a)** Radar chart of no-reference image quality score. BRISQUE and NIQE were adopted to assess the quality of benign images and malignant images in the three multi-center datasets. **(b)** Images from three datasets, the first row shows the benign cases and the second row shows the malignant cases. According to both (a) and (b) we can see dataset BUSI and BUS have a better quality than OASBUD

Table 1 summarizes the information of these datasets, the ratio of benign cases to malignant cases is imbalanced, especially for datasets BUSI and BUS. We chose blind/referenceless image spatial quality evaluator (BRISQUE) [29] and naturalness image quality evaluator (NIQE) [30] to assess the average no-reference image quality score of each dataset. Figure 1a illustrates the radar chart on these no-reference quality metrics. Note that a smaller score indicates better perceptual quality, so the dataset with a smaller bounding area has better quality, i.e., generally BUSI is better than BUS and BUS is better than OASBUD. It can be seen in Fig. 1b that with the naked eye, the images from BUSI and BUS are more distinguishable than images from OASBUD. Since the three breast ultrasound datasets above differ in countries, devices, image quality, etc., we consider them as multi-center datasets and conduct a series of experiments.

**Table 1:** Basic information of three multi-center datasets

Dataset	Center	Category	Num
BUSI	Baheya Hospital, Egypt	benign	437
		malignant	210
BUS	UDIAT Diagnostic Centre, Spain	benign	109
		malignant	54
OASBUD	Institute of Fundamental Technological Research Polish Academy of Sciences, Poland	benign	96
		malignant	104

### Preprocessing of images and graphs

**Fig. 2:**
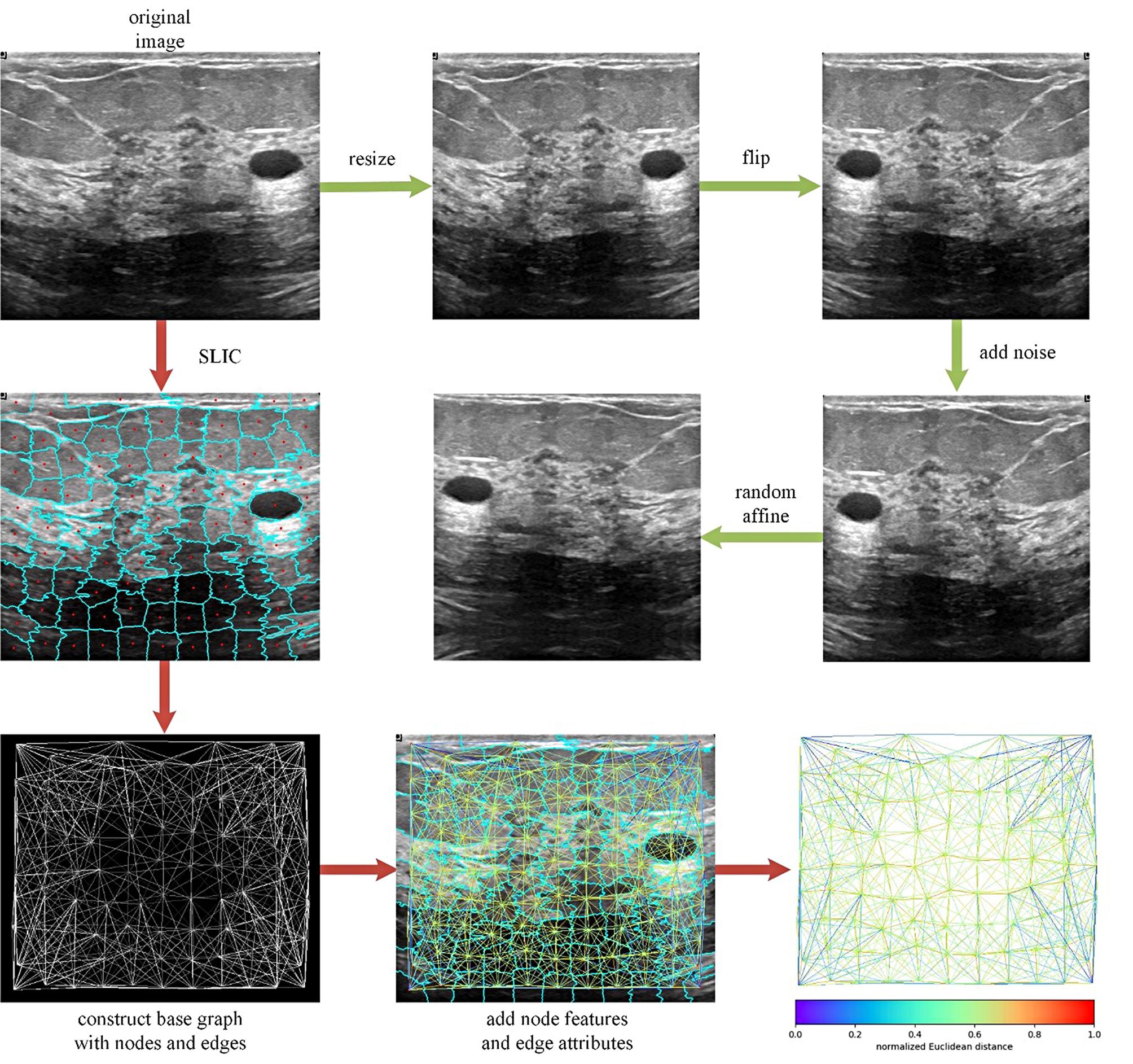
Preprocessing paths of image (green arrow) and graph (red arrow). In the green preprocessing path, the original image will be resized to N×N first, then flipped horizontally or vertically randomly and added Gaussian noise, finally applied random affine transformation. In the red preprocessing path, the SLIC algorithm will be used to segment the image based on super-pixel, then for super-pixel segments, the KNN algorithm is used to create a graph based on their centroid coordinates. Besides, according to the original image, node features are assigned by the mean value of each super-pixel segment, together with the centroid coordinate, and for each edge, the Euclidean distance is calculated as edge attributes. Finally, the complete graph is constructed, we show the edge attributes by color map here

For better training and assessing the performance of our approach on three multi-center datasets, all the images were resized to 32 × 32, then we split each dataset into training set (60%), validation set (20%) and test set (20%). Since the small amount of data, we applied augmentation methods including random horizontal/vertical flipping (probability: 50%), random affine transformation (scale: 0.9 ∼ 1.1, rotation: -3 ∼ 3 rad, shear: -3 ∼ 3 rad, translation: -0.0625 ∼ 0.0625) and adding Gaussian noise (expectation: 0, standard deviation: 0 ∼ 0.1, normal distribution), the number of training sets multiplied tenfold. Also, for better use of geometric information, we constructed graphs from images by following steps: firstly, simple linear iterative clustering (SLIC) [31], an unsupervised algorithm, converted an image to a super-pixel representation. Then, according to centroids coordinates of super-pixels, K-nearest neighbor (KNN) graphs were created. Finally, the mean value and centroid coordinate of each super-pixel segment were assigned as node features and the Euclidean distance of linked nodes was assigned as edge attribute. Table 2 summarizes the details of images and graphs in three multi-center datasets with respect to training set, validation set and test set. And Fig. 2 illustrates the paths about how to preprocess images and construct corresponding graphs.

**Table 2:** Details of images and graphs in three multi-center datasets

Dataset	Training num	Graph info	Validation num	Graph info	Test num	Graph info
BUSI	3890	∼ 120 nodes ∼ 1923 edges	129	∼ 120 nodes ∼ 1923 edges	129	∼ 120 nodes ∼ 1921 edges
BUS	1010	∼ 120 nodes ∼ 1917 edges	31	∼ 120 nodes ∼ 1915 edges	31	∼ 120 nodes ∼ 1919 edges
OASBUD	1200	∼ 119 nodes ∼ 1911 edges	42	∼ 119 nodes ∼ 1909 edges	38	∼ 120 nodes ∼ 1913 edges

### Multi-center breast tumor classification with hybrid learning

In this study, all the model architectures were built by PyTorch [32], an open-source deep learning library. The experiments were conducted on a 64-bit Ubuntu 20.04.5 long-term support operating system, equipped with a 2.50 GHz Intel(R) Xeon(R) Platinum 8255 C CPU and a NVIDIA GTX 2080Ti 11 GB GPU card.

#### Federated learning

We considered a scenario in which there are several hospitals had their own clinical data for prediction. It is a good solution to collect all the data together and train a general prediction model, however, because of privacy issues, we should keep data locally rather than exchange. Here, we proposed to use federated learning, which allows each hospital to train the model locally and keep the data safe, only the weights of models are needed for global update. We adopted FedCL [33] as the basic federated architecture, referred to the thoughts of its contrastive learning and proposed the proper loss function as follows for our task:

1 $$\eqalign{ L\,{\rm{ = }}\, & {L_{CE}}(x,\,y)\, + \,{L_{HE}}(f_{local}^{t - 1},\,f_{local}^t) \cr & + \,{L_{HE}}(f_{global}^t,\,f_{local}^t) \cr}$$

where ${L_{CE}}$ means cross entropy loss, ${L_{HE}}$ means hinge embedding loss, *x* and *y* mean classification prediction of local model and ground truth label, *f* and *t* denote the feature extracted by model and the *t*-th epoch of local training.

**Fig. 3:**
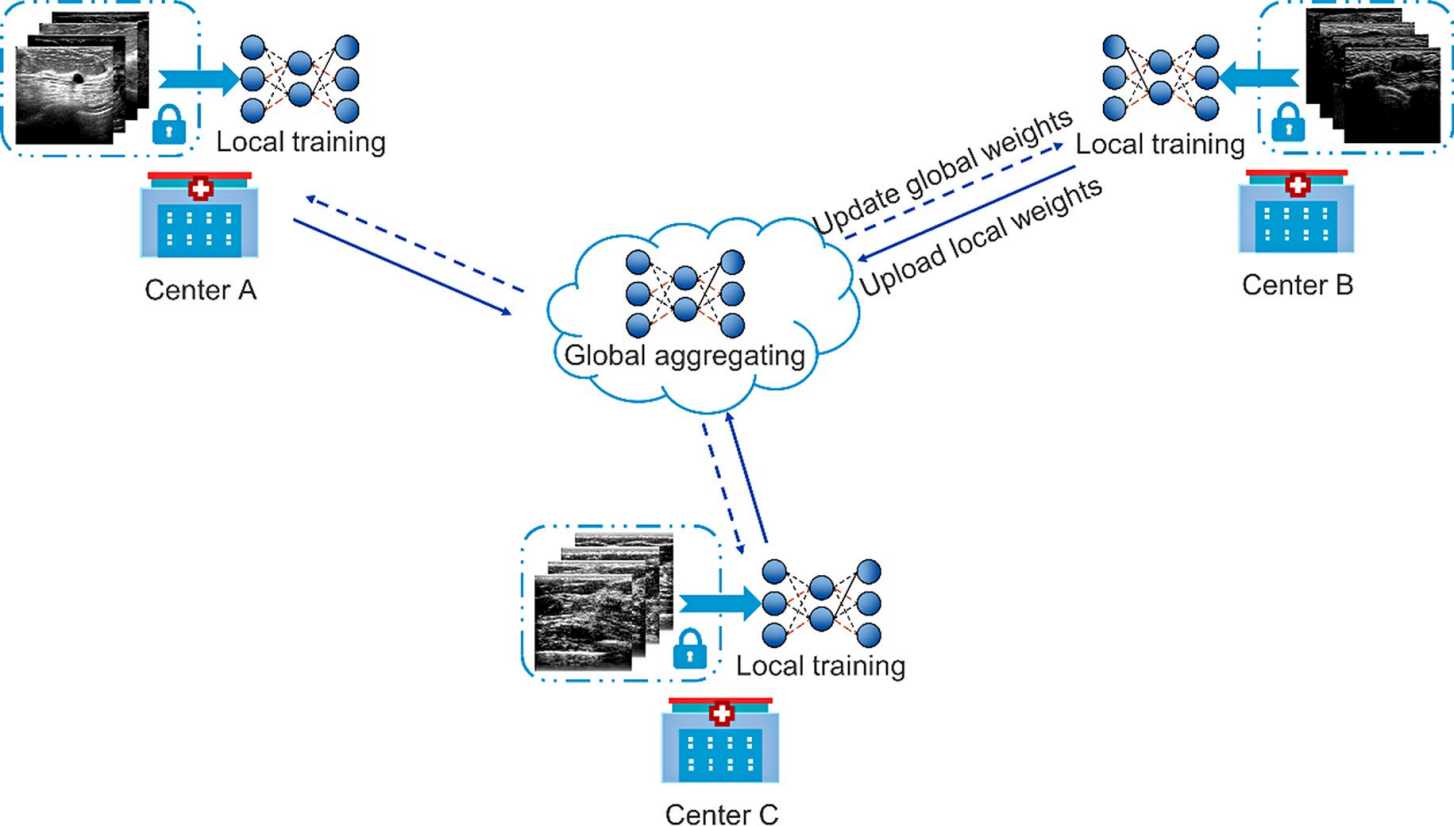
The paradigm of federated learning. The model in each center has the same architecture, it is trained with local data for several epochs and the weights will be uploaded to a public platform. Then weights of all the centers are aggregated and posted back to each center. In this way, the model can learn from multi-center datasets without data leakage

Figure 3 shows the paradigm of federated learning, each model is trained locally for several epochs before every communication round. After a certain amount of communication rounds, we considered the current local model as pretrained model for general data and it needed to fine-tune on specific data/task. In specific, the federated learning steps are as follows:

1. The center sever sends an initial global model to all the clients (Center A, Center B, Center C…), the learnable parameters of the global model are denoted as ${{\text{w}}_{glob}}$.
2. Every center trains the local model with local data for k epochs, the learnable parameters of the local model are denoted as ${w_i}(i=A,B,C...)$.
3. For each communication round, local clients send ${w_i}$ to center sever and center sever will aggregate the parameters ${{\text{w}}_{glob}} \leftarrow \sum\nolimits_{i} {\frac{{{n_i}}}{N}{w_i}}$, where ${n_i}$ is the data amount of the i-th center and N is the total number of all the data.
4. The center sever sends the updated ${{\text{w}}_{glob}}$ to local clients for next communication round.

#### Spatial and geometric learning

**Fig. 4:**
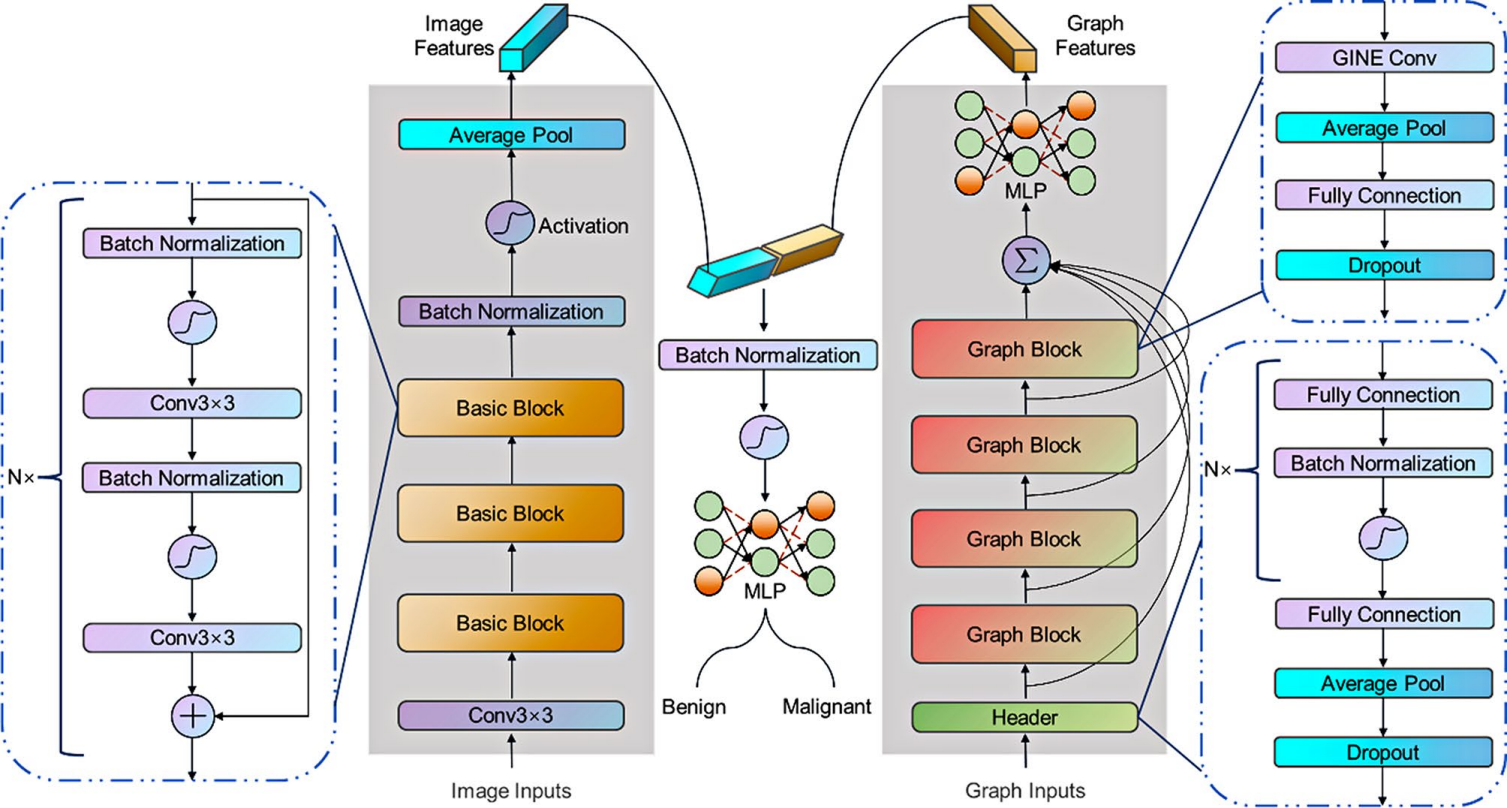
Hybrid learning architecture for breast tumor classification. The model consists of two branches, one for image features extraction and another for graph features extraction

To capture more information from the given data, we designed a two-branch model architecture which extracts features from both spatial domain and geometric domain. The details are presented in Fig. 4. In the branch of the image, we utilized PreResNet [34] as the backbone to extract features from images. It is a modified version of ResNet [35] which inherits the key operation, i.e., skip connection, and the main distinction is the order of convolution, normalization and activation. Here we adopted a depth of 110, it contained three basic blocks and each block consisted of 18 operations of skip connection. The core calculation formula of feature in PreResNet is as follows:

2 $${x_L}\, = \,{x_l}\, + \,\sum\limits_{i = l}^{L - 1} {F({x_i},{w_i})}$$

${x_L}$ and ${x_l}$ mean features of deeper unit *L* and shallower unit *l*, *F* means residual function, and *w* means weights of i-th residual unit.

Thus far, we have extracted the image features in spatial domain. In the branch of the graph, the graph isomorphism network (GIN) [36, 37] was adopted as the backbone, it consisted of four graph blocks and each block had operations of graph convolution and fully connection. Let $G=(V,E)$ denote a graph with node attributes ${X_v}(v \in V)$ and edge attributes ${e_{uv}}(u,v \in E)$(feature of edge between node *u* and *v*). The k-th layer of the representation of node *v* calculates as follows:

3 $$\eqalign{ h_v^{(k)}\, = \, & COMBIN{E^{(k)}}\,(h_v^{(k - 1)},\,AGGREGAT{E^{(k)}} \cr & (\{ (h_v^{(k - 1)},\,h_u^{(k - 1)},\,{e_{uv}}):u \in N(v)\} )) \cr}$$

Here $N(v)$ means a set of neighbors of *v*.

In addition, we added a multi-layer perceptron (MLP) with dropout to extract and refine the graph features in geometric domain. Finally, two kinds of features were concatenated and input to an MLP to make the decision whether the breast tumor was benign or malignant. Our two-branch architecture was designed to learn comprehensive information between images, node features and edge attributes, aiming to achieve better results than learning information solely.

## Results

### Metrics

In this paper, evaluation metrics were used to measure the performance of classification, including balanced accuracy, sensitivity, specificity, F1 score, AUC_ROC_ (area under the receiver operating characteristic curve), AUC_PR_ (area under the precision-recall curve). These metrics were calculated as follows:

4 $$balanced{\text{ }}accuracy=\frac{{sensitivity+specificity}}{2}$$

5 $$sensitivity=\frac{{TP}}{{TP+FN}}$$

6 $$specificity=\frac{{TN}}{{TN+FP}}$$

7 $$F1{\text{ }}score=\frac{{2 \times TP}}{{2 \times TP+FP+FN}}$$

here *TP*, *FP*, *TN* and *FN* are true positives, false positives, true negatives and false negatives respectively. Since the datasets used in this study were imbalanced, especially in BUSI and BUS, the number of benign cases was far more than malignant cases, we selected balanced accuracy to evaluate the performance. Sensitivity reveals the ability of the model to distinguish negative samples and reversely specificity reveals the ability of the model to distinguish positive samples. F1 score balances precision and recall, it reflects a comprehensive score of the model. ROC and PR curve are usually used to evaluate the classification performance and while the samples are imbalanced, the latter could reflect the differences more exactly than the former. In general, AUC is the criteria to judge which curve performs better.

### Ablation experiments of our hybrid method

**Fig. 5:**
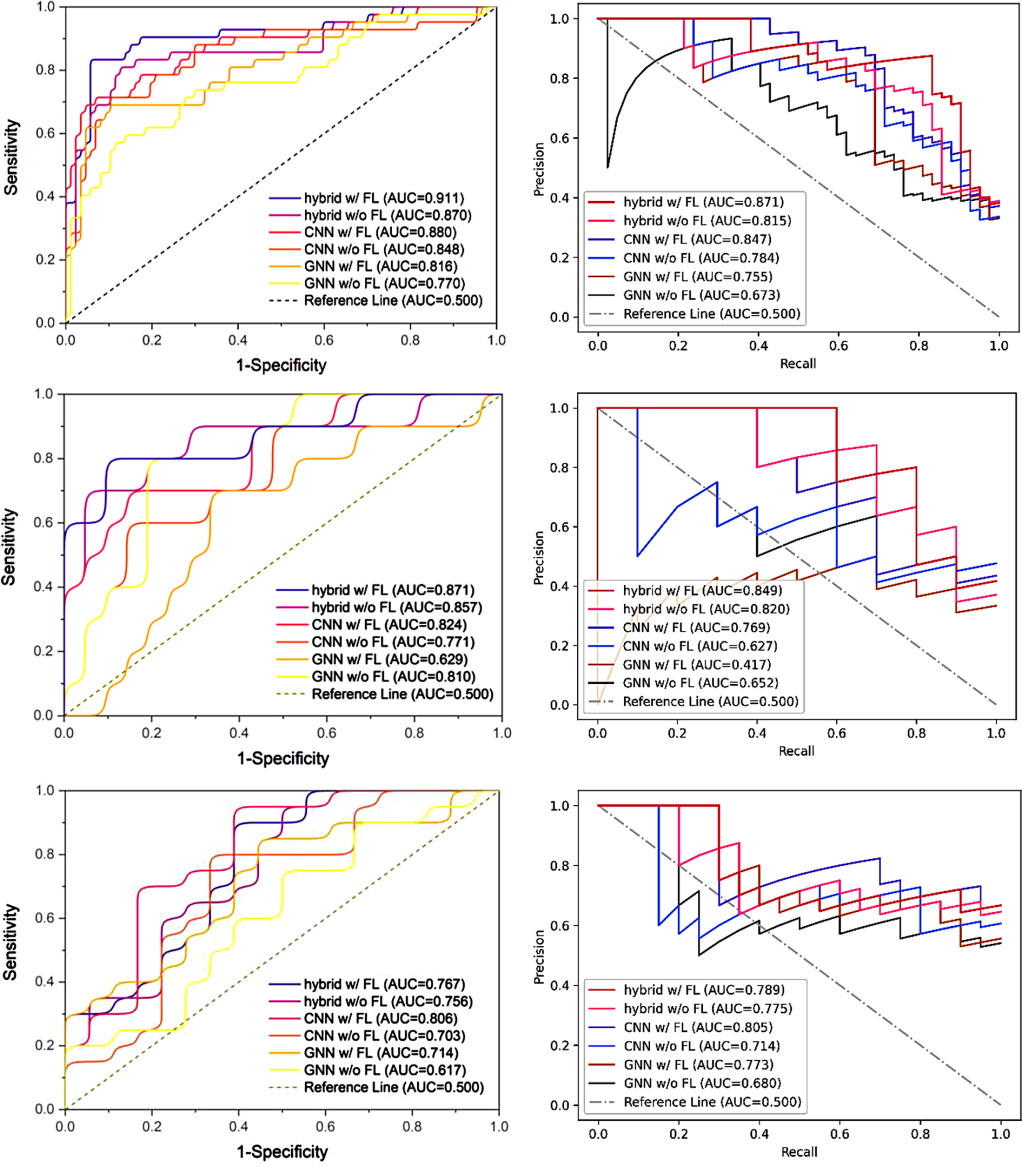
ROC (the first column) and PR curves (the second column) of all the methods. The first row, second row and third row present the curves of dataset BUSI, BUS and OASBUD respectively

We conducted a series of ablation experiments to compare the performance of models under various conditions. Our hybrid learning method, CNN method and GNN method were evaluated on three multi-center datasets with and without federated learning. The quantitative performance of all the methods were presented in Table 3, and the corresponding ROC as well as PR curves were illustrated in Fig. 5. We can see that in dataset BUSI, our method which mixed federated learning, spatial learning and geometric learning had reached best balanced accuracy of 87.6%. AUC_ROC_, AUC_PR_, F1 score and sensitivity were also the highest among all methods. For its specificity, it was the only two which exceeded 90%. As for our method without federated learning, its performance was comparable or even better to other methods with federated learning. The condition was the same in dataset BUS, our hybrid learning method with or without federated learning outperformed others with respect to almost all the metrics. However, in dataset OASBUD, the performance of all the methods slumped because of the poor quality of the images. CNN with federated learning got the best AUC_ROC_ and AUC_PR_, but that did not mean this model had a higher ability of classification since its F1 score and sensitivity were lower than 10%. Contrary to this extreme situation, our method and GNN with federated learning got relatively balanced results.

**Fig. 6:**
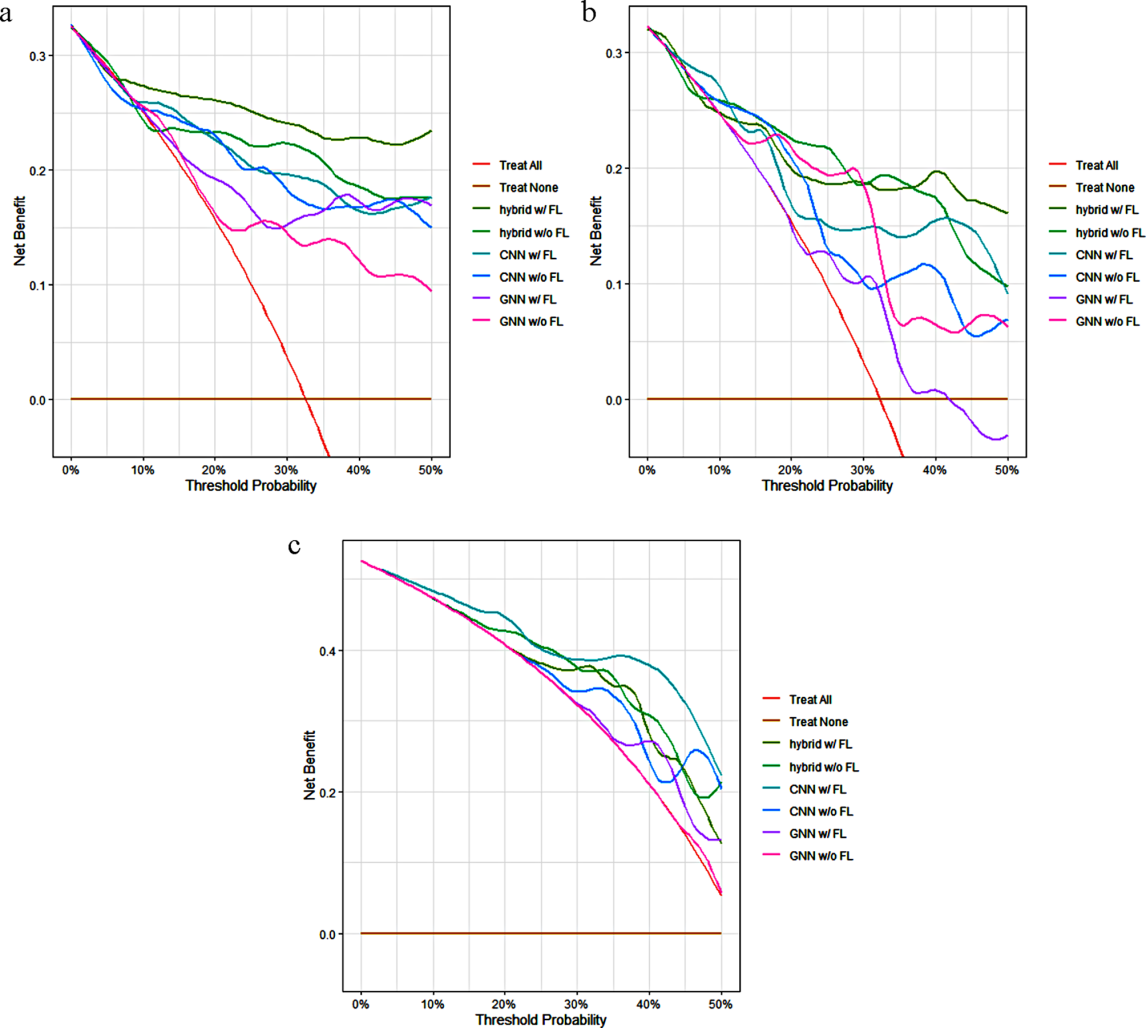
DCA of all models for three multi-center datasets. **(a)** for BUSI, **(b)** for BUS and **(c)** for OASBUD

Also, we presented decision curve analysis (DCA) of all models for three multi-center datasets in Fig. 6. For dataset BUSI and BUS, our hybrid method with or without federated learning got a relatively high net benefit than other methods in a range of threshold. In dataset OASBUD, the decision curve of CNN with federated learning seemed to perform best, like the performance of its ROC and PR curve, because of its unduly high specificity and unduly low sensitivity.

**Table 3:** Results of test sets for BUSI, BUS and OASBUD

Dataset	BUSI					
Architecture	Hybrid (ours)		CNN		GNN	
Federated learning	√	×	√	×	√	×
Balanced accuracy	**0.876**	0.811	0.782	0.805	0.788	0.729
AUC_ROC_	**0.911**	0.870	0.880	0.848	0.816	0.770
AUC_PR_	**0.871**	0.815	0.847	0.784	0.755	0.673
F1 score	**0.833**	0.753	0.706	0.741	0.716	0.633
Sensitivity	**0.833**	0.690	0.714	0.714	0.690	0.595
Specificity	0.920	**0.931**	0.851	0.897	0.885	0.862

Dataset	BUS					
**Architecture**	**Hybrid (ours)**		**CNN**		**GNN**	
**Federated learning**	**√**	**×**	**√**	**×**	**√**	**×**
Balanced accuracy	**0.852**	0.805	0.731	0.652	0.557	0.602
AUC_ROC_	**0.871**	0.857	0.824	0.771	0.629	0.810
AUC_PR_	**0.849**	0.820	0.769	0.627	0.417	0.652
F1 score	**0.800**	0.727	0.636	0.500	0.400	0.400
Sensitivity	**0.800**	0.800	0.700	0.400	0.400	0.300
Specificity	**0.905**	0.810	0.762	0.905	0.714	0.905

Dataset	OASBUD					
**Architecture**	**Hybrid (ours)**		**CNN**		**GNN**	
**Federated learning**	**√**	**×**	**√**	**×**	**√**	**×**
Balanced accuracy	0.614	**0.647**	0.525	0.544	0.619	0.544
AUC_ROC_	0.767	0.756	**0.806**	0.703	0.714	0.617
AUC_PR_	0.789	0.775	**0.805**	0.714	0.773	0.680
F1 score	**0.545**	0.500	0.095	0.308	0.483	0.308
Sensitivity	**0.450**	0.350	0.050	0.200	0.350	0.200
Specificity	0.778	0.944	**1.000**	0.889	0.889	0.889

### Performance evaluation with comparison methods

We also compared our hybrid method with four other relative state-of-the-art classification methods, including two deep learning methods for breast ultrasound and two hybrid methods for other domains. HoVer-Trans [38] was proposed based on vision transformer for breast cancer diagnosis in ultrasound images, according to the original study we resized images to 256 × 256 for model training. Another deep learning method for breast ultrasound is SBANet [17], which consisted of three training stage. Firstly, a segmentation model was trained for generating ROIs; secondly, fine-tuned the feature networks based on ROIs and origin images; finally, trained the feature aggregation network. For simplicity, we skipped the first stage and provided ground truths of ROIs directly. The images were resized to 224 × 224 according to the original study. Besides the field of breast ultrasound, we selected two more hybrid methods, FastViT [39] and MIL-ViT [40]. FastViT adopted a hybrid vision transformer architecture which used structural reparameterization to lower the memory access cost by removing skip-connections in the network, the method was able to generalize to many tasks including image classification, object detection, semantics segmentation, and 3D hand mesh regression. Hence we obtained its pretrained weights and fine-tuned for breast tumor classification task, the input size was set to 256 × 256. MIL-ViT was also a hybrid framework, combining the global semantic representation learning capability of the vision transformer and the capacity of local representation extraction from the conventional multiple instance learning. The method was for fundus image classification, we also obtained its pretrained weights and fine-tuned for our task, the input size was 384 × 384.

The comparison results are shown in Table 4. In BUSI, our method achieved best balanced accuracy, F1 score and specificity of 0.876, 0.833 and 0.920 respectively; MIL-ViT got best AUC_PR_ and specificity of 0.969 and 0.920 respectively, while SBANet got best AUC_ROC_ and sensitivity of 0.948 and 0.857 respectively. In BUS, our method performed best in more than half of the metrics (four in six). And in OASBUD, our method and Fast-ViT each got the half amount of the best results. Generally, MIL-ViT and HoVer-Trans had steady performance in every dataset while FastViT and SBANet performed poorly in BUS. FastVit and MIL-ViT had good performance in one or two datasets, while the results of HoVer-Trans were not so outstanding among three datasets.

We have calculated inference time per image (excluding the time of model initialization and weights loading) for all methods in Table 5. MIL-ViT had the fastest inference speed of only 0.007s/image, and Fast-ViT ranked second of 0.011s/image. Our hybrid model spent 0.015s. We can see that all the method just took milliseconds for prediction.

**Table 4:** Results of our hybrid method and comparative methods in BUSI, BUS and OASBUD.

Dataset	BUSI				
Method	hybrid (ours)	FastViT	MIL-ViT	HoVer-Trans	SBANet
Balanced accuracy	**0.876**	0.842	0.853	0.800	0.865
AUC_ROC_	0.911	0.855	0.938	0.854	**0.948**
AUC_PR_	0.871	0.915	**0.969**	0.890	0.915
F1 score	**0.833**	0782	0.805	0.732	0.809
Sensitivity	0.833	0.810	0.786	0.714	**0.857**
Specificity	**0.920**	0.874	**0.920**	0.885	0.874

Dataset	BUS				
Method	hybrid (ours)	FastViT	MIL-ViT	HoVer-Trans	SBANet
Balanced accuracy	**0.852**	0.729	0.776	0.755	0.650
AUC_ROC_	**0.871**	0.490	**0.871**	0.771	0.752
AUC_PR_	0.849	0.723	**0.917**	0.866	0.696
F1 score	**0.800**	0.632	0.706	0.667	0.462
Sensitivity	**0.800**	0.600	0.600	0.700	0.300
Specificity	0.905	0.857	0.952	0.810	**1.000**

Dataset	OASBUD				
Method	hybrid (ours)	FastViT	MIL-ViT	HoVer-Trans	SBANet
Balanced accuracy	0.614	**0.625**	0.519	0.589	0.597
AUC_ROC_	**0.767**	0.678	0.739	0.742	0.717
AUC_PR_	**0.789**	0.676	0.725	0.722	0.777
F1 score	0.545	**0.682**	0.591	0.500	0.667
Sensitivity	0.450	**0.750**	0.650	0.400	**0.750**
Specificity	**0.778**	0.500	0.389	**0.778**	0.444

**Table 5:** The inference time for our hybrid method and comparative methods

Method	hybrid (ours)	FastViT	MIL-ViT	HoVer-Trans	SBANet
Inference time per image(s)	0.015	0.011	0.007	0.063	0.023

## Discussion

Computer aided diagnosis have made considerable progress these years. In deep learning era, the invention of CNN made it better and faster for image processing. In this paper, we proposed to classify breast tumors by not only learning spatial features from single-center images, but also learning geometric features from corresponding graphs and hybrid features from multi-center images without data exchange.

As shown in Table 3; Fig. 5, our hybrid method with federated learning, i.e., combining all the three feature learning technologies, showed the best performance on both dataset BUSI and BUS. In dataset BUSI, its AUC_ROC_ have achieved 0.911, both sensitivity and specificity maintained a higher level than other methods. Even though without federated learning, the performance of hybrid method was still comparable or better than others. That meant the concatenation of spatial features and geometric features was more effective than single source features. For CNN, federated learning could improve the performance in terms of AUC_ROC_ and AUC_PR_. In dataset BUS, we could get the same conclusion above. Our hybrid method with federated learning outperformed all the other methods, got the highest AUC_ROC_ of 0.871, and with high sensitivity and specificity at once. But for GNN, whether in dataset BUSI or BUS, its overall performance was worse than that of CNN. A more likely reason is that in an image processing task, geometric features tend to serve as an auxiliary role rather than dominance.

It is worth note that in dataset OASBUD, the performance of ROC and PR curve was quite misleading. For example, CNN with federated learning got the best AUC_ROC_ and AUC_PR_, but its sensitivity and specificity had completely two different values. Its sensitivity of 0.050 and specificity of 1.000 meant that the model almost determined all the samples as benign, in other words it did not possess a classification ability. The extremely low F1 score also pointed out this problem. Considering all the metrics comprehensively, our hybrid method with or without federated learning as well as GNN with federated learning got relatively balanced results. In contrast with dataset BUSI and BUS, due to poor quality of dataset OASBUD, geometric features from graphs even contained more effective information than spatial features from images. That was why GNN performed better than CNN relatively.

In Table 4, we compared our hybrid method with four related state-of-the-art methods, three of them used the transformer as their backbone. Transformer was invented for sequential data, since the vision transformer was proposed, it had shown strong potentials in image processing. Different from these methods which were focusing on global and local semantic features, we focused on spatial and geometric features from images and graphs, inter- and intra- features from multicenter and local datasets. Moreover, we can see that our hybrid method performed best in some of metrics among these methods, but did not seem to have overwhelming performance compared to these state-of-the-art methods in all the metrics. However, in terms of experimental conditions, our method worked without ROIs (pixel-level labels) while SBANet needed doctors to delineate tumor contours manually; our method took only 32 × 32-sized images as input that greatly saved memory while four other models required more than 49 times larger size of images. Thus, our method still showed its advantages in the experiment.

In Table 5, we can see that two vision transformer-based methods, MIL-ViT and FastViT had shown very fast speed on inference. However, every method had took just milliseconds for prediction, in other words, they all had potentials to apply in real time medical system in the future.

There are some limitations in this study. Firstly, the method of how to construct graphs that contain richer information than now should be further studied. Secondly, the breast ultrasound datasets we used are publicly available, in the future we plan to collect and process private data from different hospitals for further multi-center research.

## Conclusions

In this paper, we proposed the hybrid learning methods for breast tumor classification, and compared with the conventional CNN and GNN. The experiments were conducted on three multi-center datasets to evaluate the performance of each model. The results confirmed the efficacy of combining spatial learning, geometric learning and federated learning. That means, spatial and geometric features, inter- and intra- features were indeed beneficial for classification task, hybrid learning had its potential in classifying breast tumors from multi-center datasets. In addition, we do not need to detect or segment breast lesions before classification, so the approach is time-consuming and directness compared to some other multi-phase methods. Our work is expected to process multi-center data without exchange and aid in the early diagnosis of the breast tumor.

## Data Availability

The data presented in this study are publicly available at https://scholar.cu.edu.eg/?q=afahmy/pages/dataset (accessed on 23 April 2024) for BUSI, http://www2.docm.mmu.ac.uk/STAFF/m.yap/dataset.php (accessed on 23 April 2024) for BUS and http://bluebox.ippt.gov.pl/~hpiotrzk/ (accessed on 23 April 2024) for OASBUD.
